# A glucose-mediated antibiotic resistance metabolic flux from glycolysis, the pyruvate cycle, and glutamate metabolism to purine metabolism

**DOI:** 10.3389/fmicb.2023.1267729

**Published:** 2023-10-17

**Authors:** Jiao Xiang, Shi-wen Wang, Yuan Tao, Jing-zhou Ye, Ying Liang, Xuan-xian Peng, Li-fen Yang, Hui Li

**Affiliations:** ^1^State Key Laboratory of Bio-Control, Southern Marine Science and Engineering Guangdong Laboratory (Zhuhai), School of Life Sciences, Sun Yat-sen University, Guangzhou, China; ^2^Laboratory for Marine Fisheries Science and Food Production Processes, Qingdao National Laboratory for Marine Science and Technology, Qingdao, China; ^3^Department of Pediatrics, The Third Affiliated Hospital of Sun Yat-sen University, Guangzhou, China; ^4^Guangdong Litai Pharmaceutical Co., Ltd., Jieyang, China

**Keywords:** antibiotic resistance, glucose, metabolic reprogramming, glycolysis, the pyruvate cycle, glutamate metabolism, purine metabolism, guanosine

## Abstract

**Introduction:**

Bacterial metabolic environment influences antibiotic killing efficacy. Thus, a full understanding for the metabolic resistance mechanisms is especially important to combat antibiotic-resistant bacteria.

**Methods:**

Isobaric tags for relative and absolute quantification-based proteomics approach was employed to compare proteomes between ceftazidime-resistant and -sensitive *Edwarsiella tarda* LTB4 (LTB4-R_CAZ_ and LTB4-S, respectively).

**Results:**

This analysis suggested the possibility that the ceftazidime resistance mediated by depressed glucose is implemented through an inefficient metabolic flux from glycolysis, the pyruvate cycle, glutamate metabolism to purine metabolism. The inefficient flux was demonstrated by the reduced expression of genes and the decreased activity of enzymes in the four metabolic pathways. However, supplement upstream glucose and downstream guanosine separately restored ceftazidime killing, which not only supports the conclusion that the inefficient metabolic flux is responsible for the resistance, but also provides an effective approach to reverse the resistance. In addition, the present study showed that ceftazidime is bound to *pts* promoter in *E. tarda*.

**Discussion:**

Our study highlights the way in fully understanding metabolic resistance mechanisms and establishing metabolites-based metabolic reprogramming to combat antibiotic resistance.

## Introduction

Antibiotic resistance is a global health challenge involving human health, food safety, and the sustainable development of animal farming. A full understanding of antibiotic resistance mechanisms is especially important to explore ways for controlling these antibiotic-resistant pathogens. Four widely accepted antibiotic resistance mechanisms include decreased membrane permeability, increased abundance of efflux pumps, hydrolase/modifying enzymes (especially beta-lactamase), and modifications of the molecular targets (Peng et al., 2019; Tong et al., 2021). Besides them, metabolism-based resistance has been recently documented (Peng et al., 2015a,b; Lopatkin et al., 2021; Fan et al., 2023; Jiang et al., 2023), indicating that metabolic adaptation may represent a class of resistance mechanisms by which cells alter their metabolic response to mitigate downstream toxic aspects of antibiotic lethality (Lopatkin et al., 2021). A metabolic state-reprogramming system can be developed to reverse antibiotic resistance (Peng et al., 2015a,b; Cheng et al., 2019; Zhao et al., 2021; Zhou et al., 2022; Chen et al., 2023; Lei et al., 2023). Therefore, further exploration of the metabolic mechanism of antibiotic resistance is helpful in developing previously unknown approaches to eliminate antibiotic-resistant pathogens (Peng et al., 2023).

Ceftazidime is a third-generation cephalosporin administered intravenously or intramuscularly. This drug has a broad spectrum of activity against Gram-positive and Gram-negative aerobic bacteria, particularly *Pseudomonas aeruginosa* and Enterobacteriaceae (including beta-lactamase-positive strains) (Richards and Brogden, 1985). However, the long-term use and wide use of ceftazidime cause the development of drug resistance in clinical isolates (Sanz-García et al., 2018; Fröhlich et al., 2022). Not only in humans but also in untreated wastewater environments and aquaculture, ceftazidime-resistant bacteria are widespread (Adelowo et al., 2018; Salgueiro et al., 2021; Moya-Salazar et al., 2022), which poses a challenge to control infection caused by the resistant pathogens in hospitals and aquaculture. Recently, we have used gas chromatography-mass spectrometry (GC-MS) to profile the metabolic state of ceftazidime-resistant *V. alginolyticus* and identified an altered and inefficient pyruvate cycle (P cycle), increased biosynthesis of fatty acids, deregulation of Na+-translocating NADH:ubiquinone oxidoreductase (Na+-NQR), and low membrane proton motive force (PMF) as the greatest metabolic modulation in the resistance (Liu et al., 2019). Meanwhile, we have also shown that intracellular reactive oxygen species (ROS) production was lower in ceftazidime-resistant LTB4 than in its isogenic ceftazidime-sensitive LTB4, which attributed ROS to inactivation of the P cycle (Su et al., 2018; Ye et al., 2018a). The resistance can be reversed by exogenous Fe^3+^, which promotes the P cycle and increases ROS production (Ye et al., 2021). These findings identify an inactivated P cycle and reduced PMF and ROS as the consequences of ceftazidime resistance. However, why the P cycle was inefficient and whether its other downstream metabolic pathways contributed to the resistance are absent.

Isobaric tags for relative and absolute quantification (iTRAQ) are powerful tools to characterize global protein changes in antibiotic-resistant bacteria (Li et al., 2018; Ye et al., 2018b). Here, the iTRAQ-based approach was used to investigate proteomes in ceftazidime-resistant and ceftazidime-sensitive *Edwarsiella tarda* LTB4 (LTB4-R_CAZ_ and LTB4-S, respectively). In combination with molecular biology and biochemistry technologies, the inefficiency of glycolysis, the P cycle, and purine metabolism was documented. This is attributed to depressed glucose. Finally, the mechanism by which glucose was depressed was explored.

## Materials and methods

### Bacterial strains and cultures

The bacteria used in the present study, *E. tarda* LTB4 and *Escherichia coli* K12 BW25113 (K12), were from the collection of our laboratory. LTB4-R_CAZ_ originated from LTB4 and was sequentially propagated in an LB medium with 1/2 MIC of ceftazidime (Ye et al., 2021). Single colonies were cultured in fresh 50 ml TSB media placed into 250 ml flasks at 30°C, with 200 rpm shaking for 24 h. The overnight cultures were collected, washed three times with saline, and suspended in M9 medium to 0.2 of OD600 nm when metabolites or/and antibiotics were added if desired. These bacterial cells were cultured at 37°C (K12) or 30°C (LTB4 and LTB4-R_CAZ_) at 200 rpm for 6 h.

### Sample preparation and iTRAQ labeling quantitative proteomics

Sample preparation and iTRAQ labeling analysis were described previously without any modifications (Ye et al., 2018a). In brief, 100 μg of proteins per sample were reduced by 200 mM Tris (2-carboxyethyl) phosphine and alkylated with 25 mM iodoacetamide. These proteins were digested by Promega trypsin and labeled with iTRAQ labeling kits. Liquid chromatography-tandem/mass spectrometry (LC-MS/MS) was adopted and performed on an AB SciexTripleTOF 5600 mass spectrometer (AB SCIEX; Concord, ON, Canada) with a NanoAcquity UPLC (ultraperformance liquid chromatography) (Waters, Milford, MA, USA) system. All LC–MS/MS data were processed using ProteinPilot version 4.5 software (Applied Biosystems, USA). Protein identification was performed by the MS/MS data searching against the *E. tadar* EIB202 database with at least two peptides matched. Differential abundances of proteins were selected using iTRAQ average reporter ion ratio threshold values of ≥1.5 (increased) or ≤ 0.667 (decreased), with both p-values between two biological replicates <0.05. Two biological repeat correlations were analyzed using IBM SPSS Statistics 19 software. The differential expression of proteins in the GO annotation and KEGG pathways was analyzed with OmicsBean (http://www.omicsbean.cn/). The functions of each protein were categorized according to the GO annotation based on the biological process hierarchy. The protein-protein interaction network was implemented using STRING and Cytoscape software. iPath analysis was carried out using a web-based tool (http://pathways.embl.de) for the visualization and analysis of cellular pathways.

### Measurement of enzymatic activity

The activity of glucokinase (HK), 6-phosphofructokinase I (PFK), pyruvate kinase (PK), and glutamic-pyruvic transaminase (GPT) was measured according to kit instructions (Solarbio, Beijing, China). The activity of pyruvate dehydrogenase (PDH), α-ketoglutarate dehydrogenase (KGDH), succinate dehydrogenase (SDH), and malate dehydrogenase (MDH) was detected as described previously (Cheng et al., 2018). In brief, bacteria were cultured in an M9 medium with or without glucose at 30°C with shaking at 200 rpm for 6 h. Cells were collected, immediately snap-frozen in liquid nitrogen, and stored at−80°C. The cells were resuspended with 1 ml of PBS and disrupted by sonic oscillation for 10 min (200 W total power with 35% output, 2 s pulse, and 3 s pause over ice). The protein concentration of the supernatant was quantified with a bicinchoninic acid (BCA) kit (Beyotime, P0009, China), and the final protein concentration for each sample was diluted to the same concentration. The activity of glucokinase (HK), 6-phosphofructokinase I (PFK), and pyruvate kinase (PK) was determined at 340 nm using commercial assay kits (Solarbio, China; BC0745, BC0535, and BC0545, respectively). The activity of glutamic-pyruvic transaminase (GPT) was determined at 505 nm using a glutamic-pyruvic transaminase activity assay kit (Solarbio BC1555, China). For measurement of PDH and KGDH, a reaction mixture containing 0.15 mM 3-(4,5-dimethyl-2-thiazolyl)-2,5-diphenyl- 2H-tetrazolium bromide (MTT), 2.5 mM MgCl_2_, 6.5 mM phenazine methosulfate (PMS), 0.2 mM thiamine PPi (TPP), and 80 mM sodium pyruvate/alpha-ketoglutaric acid potassium salt was used. Another reaction mixture containing 0.15 mM MTT, 2.5 mM MgCl_2_, 13 mM PMS, and 80 mM sodium succinate/sodium malate was used for SDH and MDH activity measurement. Then, the reaction mixtures were incubated at 37°C for 30 min for MDH, PDH, and KGDH and 5 min for SDH and detected at 562 nm for colorimetric readings. Absorbance was measured using a microplate reader (Epoch2, BioTek Instruments, Inc., United States). All experiments were normalized by the protein concentration. Experiments were repeated in at least three independent biological replicates.

### Quantitative real-time PCR

To investigate the effect of glucose on gene expression levels, quantitative real-time PCR (qRT-PCR) was performed as previously described (Cheng et al., 2017). In brief, after incubation in M9 with or without glucose at 30°C with 200 rpm for 6 h, the cells were collected and adjusted to 1.0 OD600 nm. Total RNA was isolated from 1 ml cell samples using TRIzol reagent (Invitrogen Life Technologies). cDNA was obtained from 1 μg of total RNA, and reverse transcription was performed according to a PrimeScript RT reagent kit with gDNA Eraser (Guangzhou IGE Biotechnology Ltd., China). The primers used for qRT-PCR are listed in Supplementary Table 1, where the 16S rRNA gene served as an internal control. The qRT-PCR was performed in 384-well plates with the SYBR Green Premix Pro Taq HS qPCR Kit (Guangzhou IGE Biotechnology Ltd., China) at a total volume of 10 ml. The reaction mixtures were run on a LightCycler 480 system (Roche, Germany). The cycling parameter values were set as follows: 95°C for 30 s to activate the polymerase, 40 cycles of 95°C for 5 s, and 58°C for 30 s. Fluorescence measurements were performed at 75°C for 1 s during each cycle. Cycling was terminated at 95°C with a calefactive velocity of 0.11°C s^−1^ to obtain a melting curve. Data were calculated as relative mRNA expression compared to the glucose group without the endogenous reference 16S rRNA gene.

### Antibiotic-bactericidal assay

As previously described, an antibiotic bactericidal assay was performed (Jiang et al., 2020) in M9 with or without metabolites and/or antibiotics and incubated at 30°C with 200 rpm. After 6 h, 100 μl of samples was 10-fold continuously diluted, and 5 μl of each dilution was aliquoted on the 2% TSB agar plates and cultured at 30°C for ~24 h, and CFU per ml was determined. The percent survival was determined by dividing the CFU obtained from the treated sample by the CFU obtained from the control.

### Measurement of glucose content

A series of experiments was performed according to the kit instructions (Solarbio, China; BC2505). In brief, bacteria were incubated in M9 with or without glucose at 30°C with 200 rpm for 6 h. Cells were collected and adjusted to 1.0 OD600 nm for subsequent assays. An aliquot of 10 ml cell samples was disrupted by sonic oscillation for 10 min (200 W total power with 35% output, a 2 s pulse, and a 3 s pause over ice). A portion of the supernatant was used to determine protein content using a bicinchoninic acid (BCA) kit (Beyotime, P0009, China). At the same time, the others were used for 505 nm absorbance measurement using a microplate reader (Epoch2, BioTek Instruments, Inc., United States). All experiments were normalized by the protein concentration.

### Binding of ceftazidime to *pts* promoter

As previously described (Jiang et al., 2023), PCR products of *pts* promoter fragments were purified using a PCR purification kit (Sangon Biotech, Shanghai, China), and their concentration was quantified. Various concentrations of DNA, ranging from 100 to 3,200 ng, were mixed with 10 μl of ceftazidime with a concentration of 100 mg/ml, and finally, the total volume was made up to 100 μl with Tris–HCl buffer at 37°C for 3 h. A total of 20 tubes were pooled and precipitated by 1:1 isopropanol for 10 min and centrifuged at 14,000 *g* for 10 min. The precipitation was washed three times with 75% ethanol and dissolved in 10 μl of Tris–HCl buffer for subsequent assays. Overnight *Escherichia coli* K12 cultures were collected and washed with a saline solution. The cultures were adjusted to an OD600 of 0.6 with M9 medium and diluted to 1:100,000. Then, 50 μl of bacteria was incubated with or without *pst* promoter precipitated by isopropanol as described above at 30°C with shaking at 200 rpm for 6 h. CFU per milliliter was determined as described above.

## Results

### Differential abundances of proteins identified by itraq labeling technology

To understand *E. tarda* resistance to ceftazidime at the protein level, LTB4-R_CAZ_ was generated from LTB4-S. There was a 16-fold difference between LTB4-R_CAZ_ and LTB4-S (Supplementary Figure 1A). Protein samples of the two strains were analyzed using iTRAQ proteomics technology. In total, 2,013 proteins were identified in each sample. Linear correction was calculated with 0.81 between all proteins in LTB4-S and 0.911 between all proteins in LTB4-R_CAZ_ (Supplementary Figure 1B), suggesting the high quality of the iTRAQ analysis. Among the 2,013 proteins, 150 showed differential abundances. Among them, 91 proteins were upregulated and 59 were downregulated (Supplementary Tables 2, 3).

**Figure 1:**
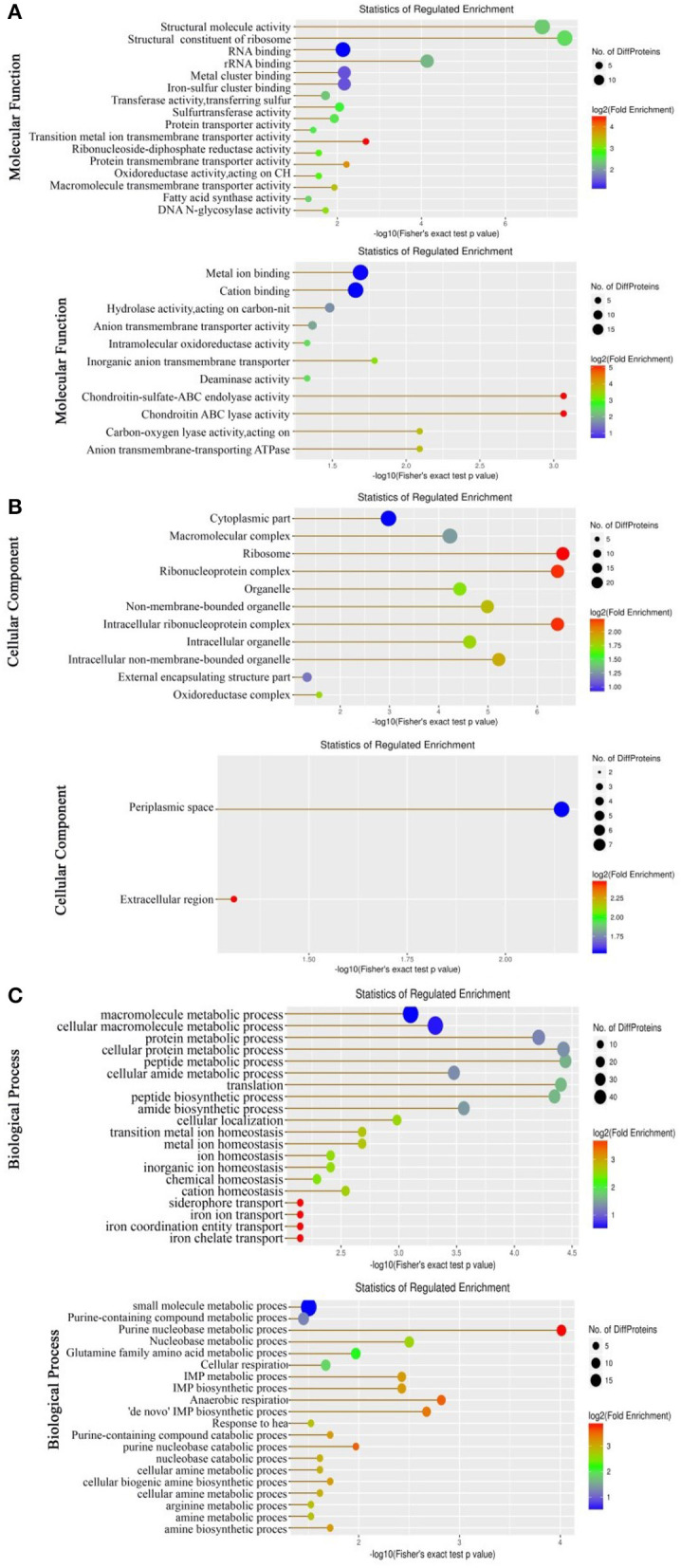
Molecular functional **(A)**, cellular component **(B)**, and biological process **(C)** of the differential abundance of proteins using GO (Gene Ontology) analysis. Up, Upregulation proteins. Down, Downregulation proteins. In biological processes, there are 10 pathways upregulated that are related to iron ions. Proteins in these 9 pathways were NDH, ETAC_08255 (HMUS), FECA, and ETAC_08250 (outer membrane receptor for ferrienterochelin and colicins). These four proteins cannot enrich any KEGG pathway.

Gene Ontology (GO) enrichment analysis was carried out on the upregulated and downregulated expressed proteins to classify molecular functions, biological processes, and KEGG pathways. For the analysis of molecular functions, proteins that were upregulated and downregulated were enriched in 16 and 11 genes, respectively. Almost all of them are involved in molecular activity and binding (Figure 1A). For the analysis of cellular components, more cellular components were documented in the upregulated proteins than in the downregulated proteins. Specifically, 11 were enriched (mostly belonging to organelles and protein complexes) in the upregulated proteins, while 2 (periplasmic space and extracellular region) were found in the downregulated proteins (Figure 1B). For the analysis of biological processes, equal numbers (20) were enriched in both the upregulated and downregulated proteins, but the involved biological processes were differential. The biological processes enriched by the upregulated proteins mostly belong to metabolic processes, ion homeostasis, and iron transport, whereas the biological processes enriched by the downregulated proteins mostly contribute to metabolic and catabolic processes. Although the metabolic processes of the general and detailed items overlap, they are not the same. Specifically, the enriched metabolic processes in the upregulated proteins mostly include macromolecule, protein, and peptide metabolic processes, whereas those in the downregulated proteins mostly contain nucleobase and amino acid metabolic processes, where, importantly, most contribute to purine metabolism (Figure 1C). These results indicate that a global change is determined in LTB4-R_CAZ_, where downregulated purine metabolism is the most characteristic feature of the strain.

### Protein-protein interaction analysis and pathway enrichment

**Figure 2:**
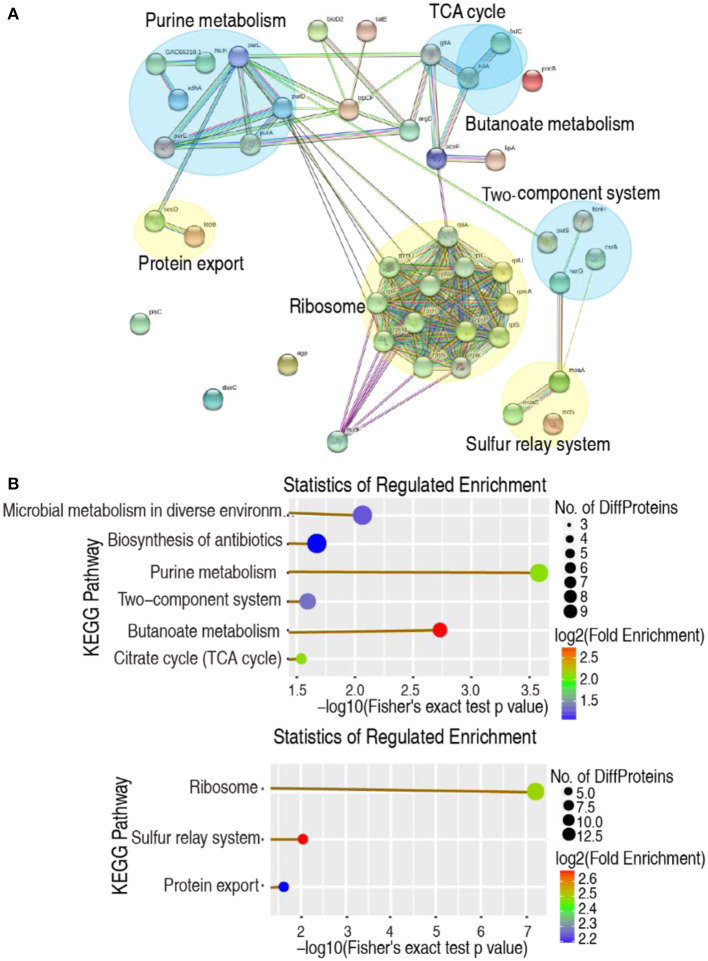
Functional proteins associated with metabolism. **(A)** The predicted physical and functional network of protein–protein interaction. Yellow, Upregulation pathway. Blue, Downregulation pathway. **(B)** KEGG function enrichment of metabolic pathway. Upper, Downregulation pathway. Lower, Upregulation pathway (company give-out).

Protein-protein interaction (PPI) can help study the biological pathways and signal networks of cells (Ayub and Naveed, 2022). The 150 differential abundances of proteins were used to construct a PPI network using the web-based tool STRING. Among them, 53 were matched in the databases, resulting in the creation of a network based on seven functional categories, where 3 and 4 were upregulated and downregulated, respectively. Specifically, the ribosome, sulfur relay system, and protein export were upregulated, whereas purine metabolism, butanoate metabolism, the two-component system, and the TCA cycle were downregulated (Figure 2A). These were consistent with the data from the KEGG pathway analysis, except for the more reduced microbial metabolism in diverse environments and the biosynthesis of antibiotics in the KEGG pathway analysis (Figure 2B). Therefore, decreased glucose causes the negative modulation of central carbon metabolism and its downstream nucleotide metabolism, especially purine metabolism.

### Global overview and key metabolic pathway summary

**Figure 3:**
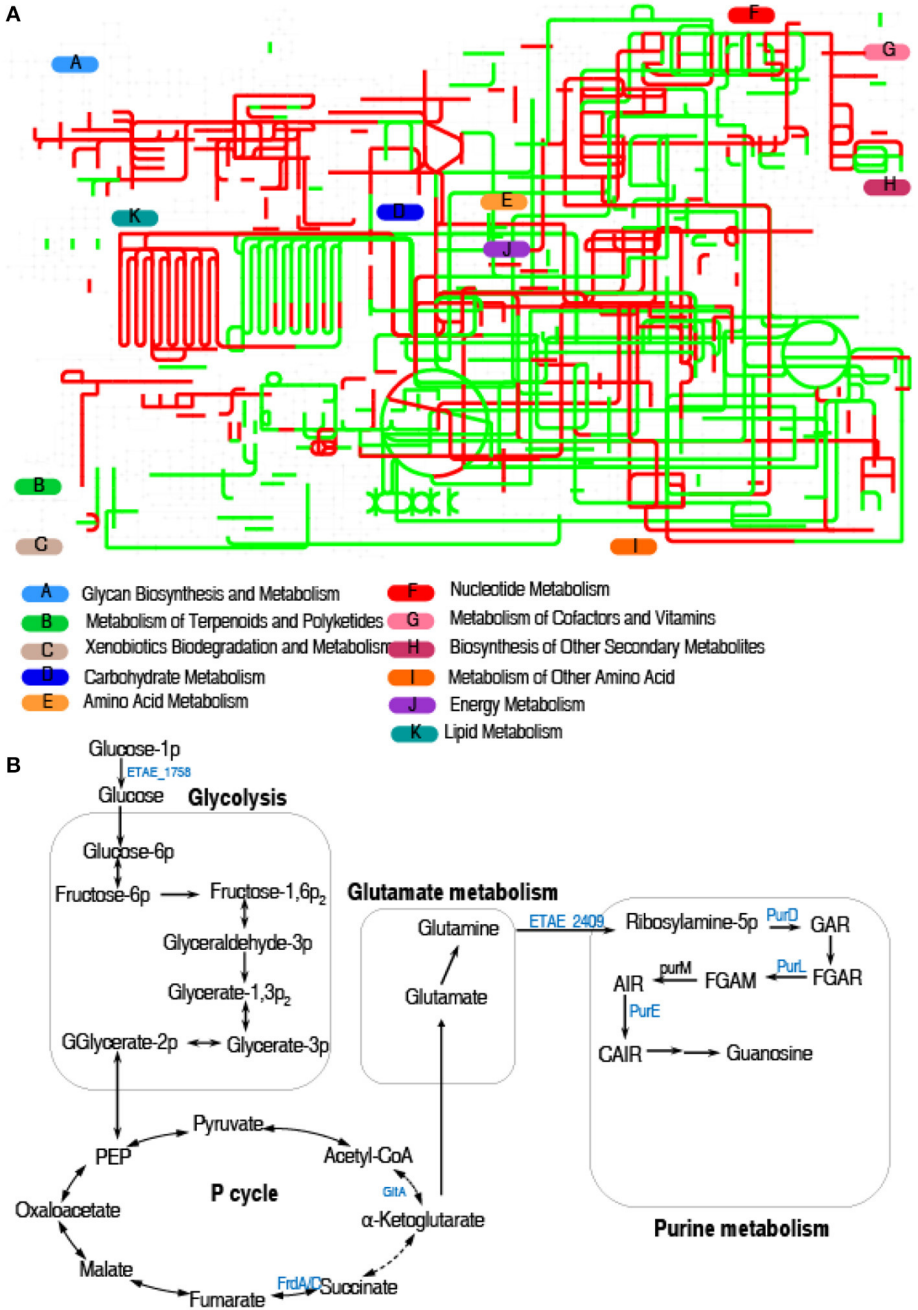
Metabolic pathway analysis. **(A)** Analysis for metabolic profiling showed a better insight into the effects of differential abundance proteins (*p* < 0.05). Based on the EggNOG database (http://eggnog5.embl.de), metabolic network pathways are further analyzed using iPath2.0 (http://pathways.embl.de). Red, increase; green, decrease. **(B)** Metabolic schematic diagram of differential proteins. The identified downregulated proteins (green) involved in metabolism are all shown in the flowchart.

Interactive Pathways Explorer (iPath) is a web-based tool for the visualization, analysis, and customization of various pathway maps (Darzi et al., 2018). Therefore, we used iPath to display a comparative global pathway analysis between LTB4-S and LTB4-R_CAZ_ for a better insight into the effects of ceftazidime resistance on global metabolism. As shown in Figure 3A, red and green lines represent upregulated and downregulated pathways, respectively, in LTB4-R_CAZ_. Generally, carbohydrate metabolism, energy metabolism (especially the TCA cycle), and nucleotide metabolism were reduced. Furthermore, the mainly altered metabolism caused by the downregulated proteins is outlined in Figure 3B. These results suggest that inefficient glycolysis, the P cycle, and purine metabolism may be responsible for the resistance, which is related to the reduction of glucose.

### Inactivation of glycolysis and the P cycle

**Figure 4:**
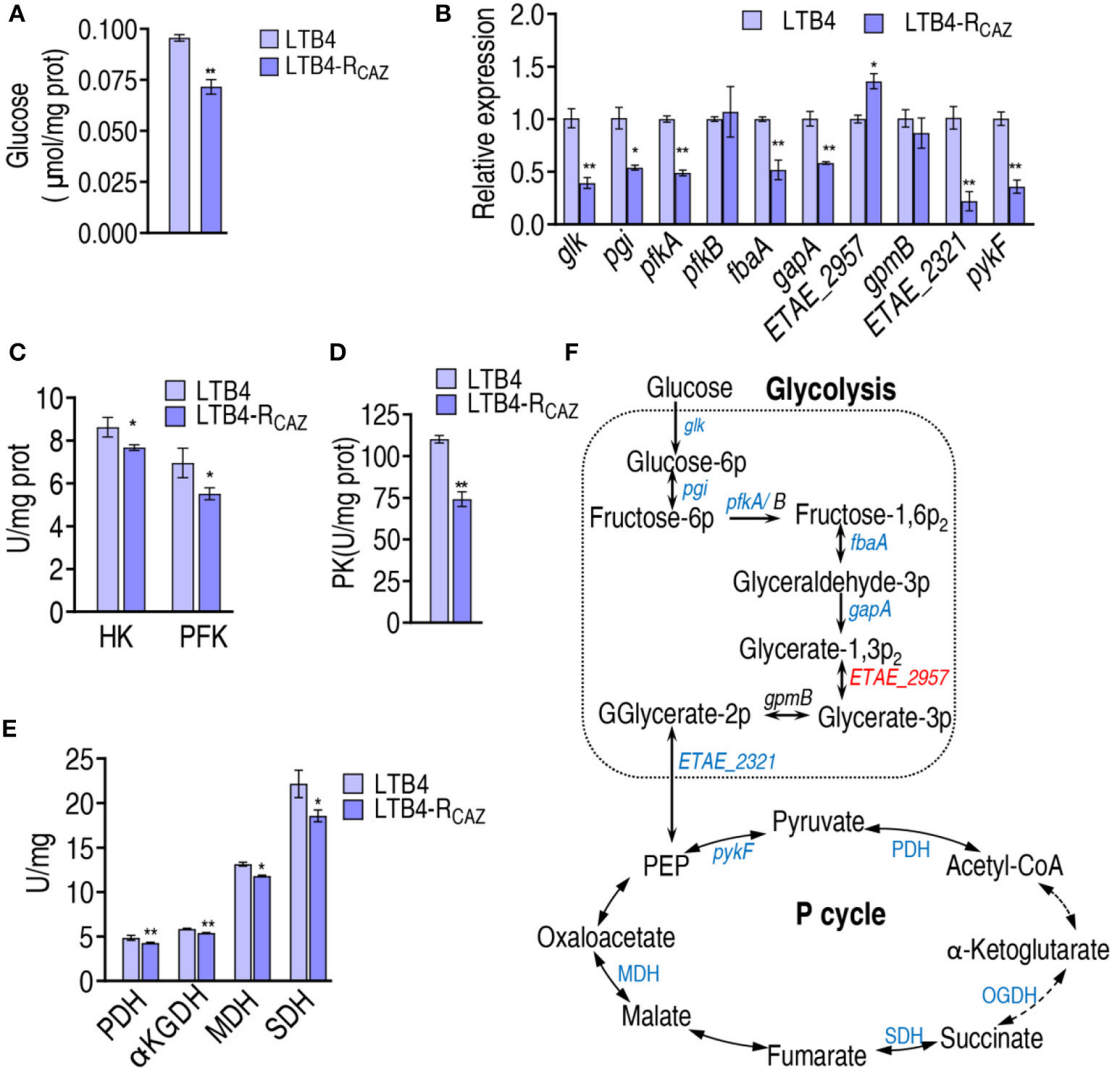
Alteration of glycolysis and the P cycle. **(A)** Quantification of glucose in LTB4-S and LTB4-R_CAZ_. **(B)** qRT-PCR for expression of genes encoding glycolysis and *pykF* in the P cycle. **(C)** Activity of HK and PFH in glycolysis. **(D)** Activity of PK in the P cycle. **(E)** Activity of enzymes in the P cycle. **(F)** A summarizing map showing the alteration of glycolysis and the P cycle. ^*^, *P* < 0.05; ^**^, *P* < 0.01.

To confirm the above findings on the inactivation of glycolysis and the P cycle that is attributed to the reduced glucose, glucose content, qRT-PCR, and enzyme activity measurements were performed in LTB4-S and LTB4-R_CAZ_. Lower glucose was measured in LTB4-R_CAZ_ than in LTB4-S (Figure 4A). qRT-PCR was employed to detect nine genes encoding glycolysis. Among them, *glk, pgi, pfkA, fbaA, gapA*, and ETAE_2321 exhibited decreased expression, and the other three, ETAE_2957, *pfkB*, and *gpmB*, displayed increased and unchanged expression in LTB4-R_CAZ_ compared with LTB4-S (Figure 4B). Further measurement of enzyme activity in glycolysis showed lower activity of glucokinase (HK, encoded by *glk*) and 6-phosphofructokinase I (PFK, encoded by *pfkA* and *pfkB*) in LTB4-R_CAZ_ than LTB4-S (Figure 4C). Since the reduction of gene expression in the P cycle was reported in our previous report (Ye et al., 2021), only *pykF* was selected as a representative gene to confirm the reduction. Expression of *pykF* was lower in LTB4-R_CAZ_ than in LTB4-S (Figure 4B). Consistently, pyruvate kinase (PK, encoded by *pykF*) was lower in LTB4-R_CAZ_ compared with that in LTB4-S (Figure 4D). The activity of pyruvate dehydrogenase (PDH), α-ketoglutarate dehydrogenase (KGDH), succinate dehydrogenase (SDH), and malate dehydrogenase (MDH) was lower in LTB4-R_CAZ_ than in LTB4-S (Figure 4E). Therefore, glycolysis and the P cycle were inefficient due to the depressed glucose (Figure 4F).

### Inactivation of glutamate metabolism and purine metabolism

**Figure 5:**
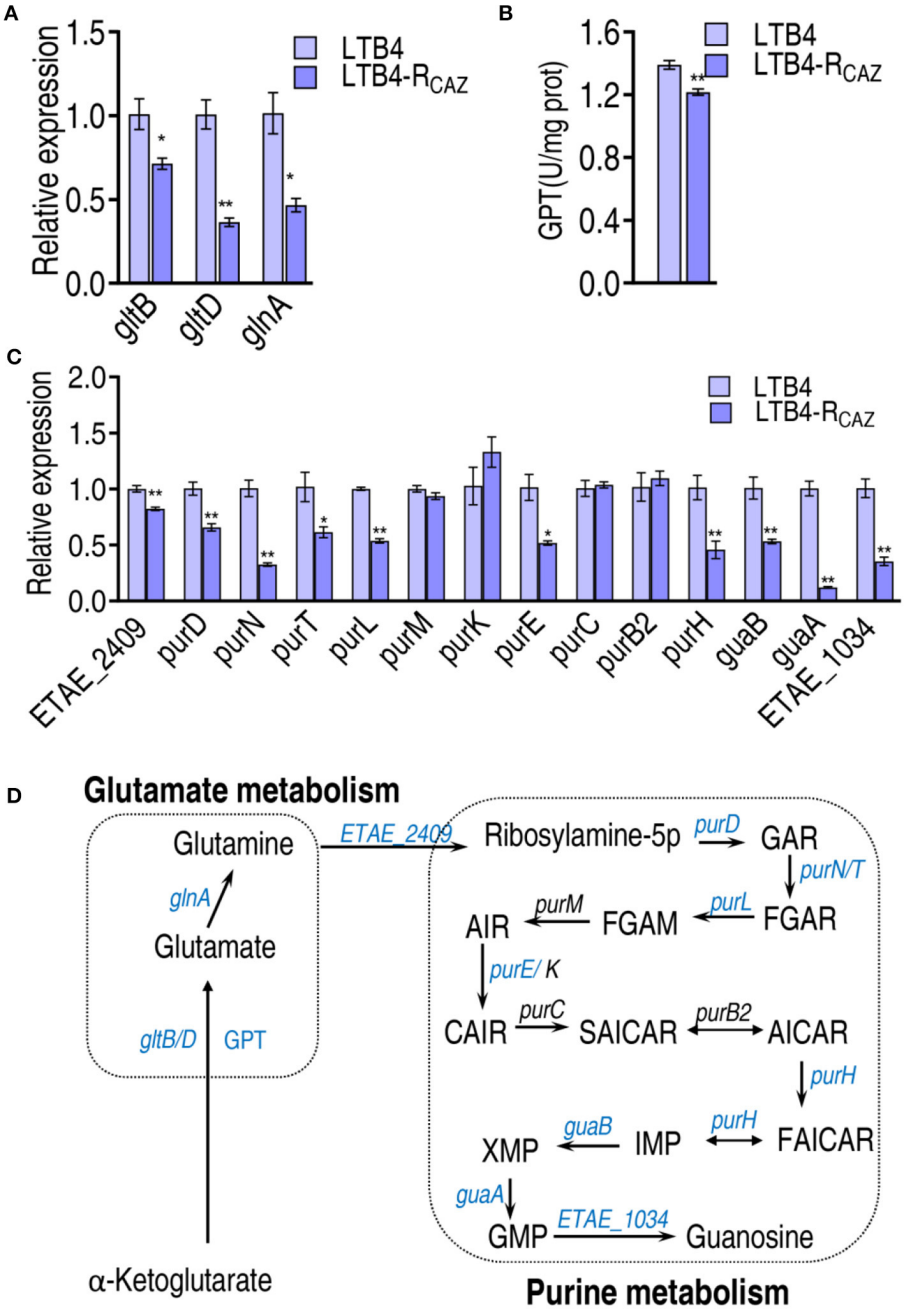
Alteration of glutamine metabolism and purine metabolism. **(A)** qRT-PCR for expression of genes encoding glutamate metabolism. **(B)** Activity of alanine transaminase (GPT). **(C)** qRT-PCR for expression of genes encoding purine metabolism. **(D)** A summarizing map showing the alteration of glutamate metabolism and purine metabolism. ^*^, *P* < 0.05; ^**^, *P* < 0.01.

The P cycle metabolism fluxes to purine metabolism *via* glutamate metabolism. The expression of *gltD* and *gltB* (encoding glutamate synthases) and *glnA* (catalyzing the ATP-dependent biosynthesis of glutamine from glutamate and ammonia) was reduced in LTB4-R_CAZ_ (Figure 5A). Similarly, the activity of glutamic-pyruvic transaminase (GPT) converting 2-oxoglutarate to glutamate was decreased in LTB4-R_CAZ_ (Figure 5B). Following the measurement of glutamate metabolism, the expression of genes encoding purine metabolism was quantified. A total of 14 genes were measured. Among them, 10 (ETAE_2409, *purD, purN, purT, purl, purE, purH, guaB, guaA*, and ETAE_1034) and 4 (*purM, purK, purC*, and purB2) displayed lower and unchanged expression in LTB4-R_CAZ_ than LTB4-S, respectively (Figure 5C). These results confirm the inactivation of glutamate metabolism and purine metabolism (Figure 5D).

### Role of glucose in the flux from glycolysis to purine metabolism

**Figure 6:**
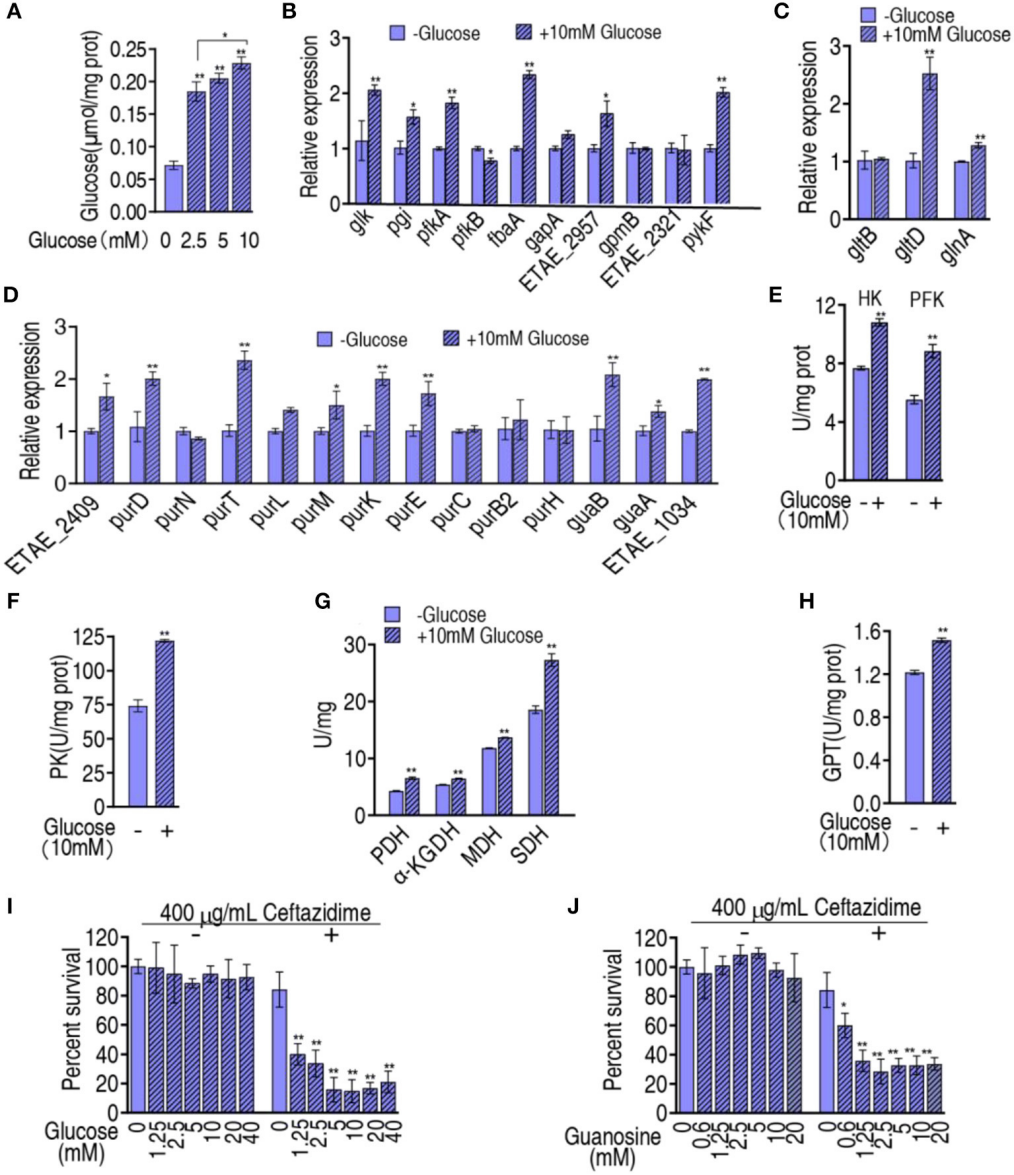
Effect of exogenous glucose on glycolysis, the P cycle, glutamate metabolism, and purine metabolism. **(A)** Intracellular glucose of LTB4-R_CAZ_ in the presence of the indicated concentrations of exogenous glucose. **(B–D)** qRT-PCR for expression of genes encoding glycolysis, the P cycle **(B)**, glutamate metabolism **(C)**, and purine metabolism **(D)**. (**E–H)**, Activity of enzymes in glycolysis **(E)**, the P cycle **(F, G)**, glutamate metabolism. **(I)** Survival of LTB4-R_CAZ_ in the presence of the indicated concentration of glucose plus ceftazidime. **(J)** Survival of LTB4-R_CAZ_ in the presence of the indicated concentration of guanosine. ^*^, *P* < 0.05; ^**^, *P* < 0.01.

The above results motivated us to suppose that the reduced glucose is responsible for the inefficient flux from glycolysis, the P cycle, and glutamate metabolism, to purine metabolism. To do this, exogenous glucose was complemented to test whether the inefficient flux was reversed. When glucose was added, the intracellular glucose of LTB4-R_CAZ_ was elevated in a glucose dose-dependent manner (Figure 6A). Then, qRT-PCR was used to measure the expression of the 9 genes of glycolysis, the 1 gene (*pykF*) of the P cycle, the 3 genes of glutamate metabolism, and the 14 genes of purine metabolism in LTB4-R_CAZ_ cultured in medium with and without glucose. Exogenous glucose promoted 6 genes of glycolysis, 1 gene of the P cycle, 2 genes of glutamate metabolism, and 9 genes of purine metabolism. The others kept stable except for weakly reduced *pfkB* (Figures 6B–D). Consistently, the activity of HK and PFK of glycolysis, PK, PDH, KGDH, and SDH of the P cycle, and GPT of glutamate metabolism were also elevated (Figures 6E–H). To test whether glucose reversed the ceftazidime resistance, the viability of LTB4-R_CAZ_ was measured in the presence of different concentrations of glucose plus ceftazidime. The survival was reduced in a glucose dose-dependent manner between 1.25 mM and 5 mM glucose and kept stable between 5 mM and 40 mM (Figure 6I). Guanosine is an intermetabolite of purine metabolism that was repressed in LTB4-R_CAZ_ (Liu et al., 2019). Thus, glucose was replaced with guanosine to explore whether the intermetabolite has the potential. A similar result was determined in the replacement (Figure 6J). Therefore, glucose reduction can cause guanosine-mediated antibiotic resistance through the metabolic flux from glycolysis, the P cycle, and glutamate metabolism to purine metabolism.

### Mechanisms by which glucose was reduced

**Figure 7:**
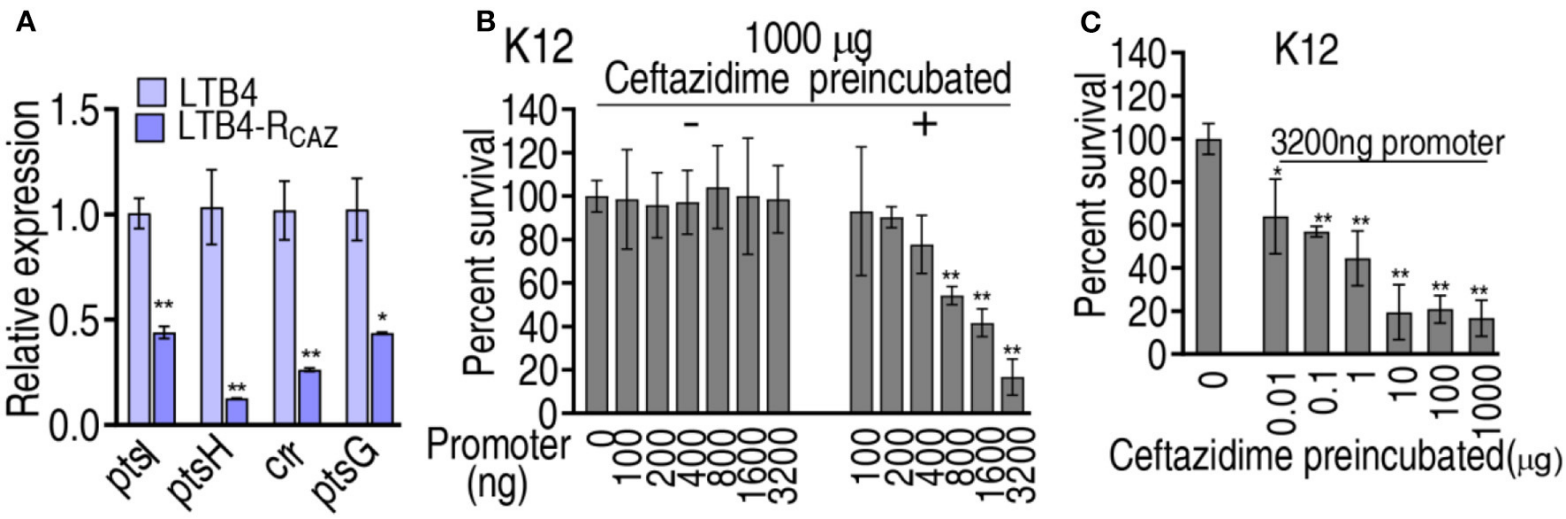
Expression of genes controlled by the *pts* promoter and the binding of *pts* promoter with ceftazidime. **(A)** qRT-PCR for expression of genes controlled by the *pts* promoter in LTB4-S and LTB4-R_CAZ_. **(B)** Survival of *E. coli* K12 exposed to ceftazidime isolated from the binding of 1,000 μg ceftazidime to the indicated concentration of *pts* promoter. **(C)** Survival of *E. coli* K12 exposed to ceftazidime isolated from the binding of 3,200 μg *pts* promoter with the indicated concentration of ceftazidime. ^*^, *P* < 0.05; ^**^, *P* < 0.01.

Our recent report has revealed that ampicillin binds with the *pts* promoter to negatively regulate the PTS system encoded by *ptsL, ptsH, crr*, and *ptsG*, which downregulates the glucose levels in antibiotic-resistant *E. coli* (Jiang et al., 2023). Ampicillin and ceftazidime belong to the same class of antibiotic β-lactam. Thus, we supposed that ceftazidime may have the same ability that binds to *pts* promoter. To explore this idea, qRT-PCR was used to detect the expression of *ptsL, ptsH, crr*, and *ptsG*. Lower expression of the four genes was quantified in LTB4-R_CAZ_ than in LTB4-S (Figure 7A). To test whether the reduced expression of the four genes was also attributed to the binding of ceftazidime with the *pts* promoter, ceftazidime was incubated with different concentrations of *pts* promoter. The bound ceftazidime was used to kill *E. coli* K12. The viability of *E. coli* K12 was reduced in a *pts* promoter dose-dependent manner (Figure 7B). Meanwhile, the *pts* promoter was incubated with different concentrations of ceftazidime to capture ceftazidime and then used for an antibacterial test. Survival of *E. coli* K12 was decreased with increasing ceftazidime between 0.01 μg and 10 μg and kept stable between 10 μg and 1,000 μg (Figure 7C). These results are consistent with the finding that antibiotics control *pts* promoters to manipulate glucose-mediated antibiotic resistance (Jiang et al., 2023).

## Discussion

Metabolic environments are changed to respond to antibiotic resistance, as has been documented (Stokes et al., 2019; Zhang et al., 2019; Li et al., 2020; Chen et al., 2022b), where intermetabolites of purine metabolism, such as inosine and guanosine, are reduced (Liu et al., 2019; Zhao et al., 2021). However, information regarding the reduction mechanisms is not clarified. The present study employs a proteomics approach to characterize the differential abundance of proteins between LTB4-R_CAZ_ and LTB-S. This leads to the possibility that ceftazidime depresses glucose and the depressed glucose causes the reduction of purine metabolism through a glucose-mediated metabolic flux from glycolysis, the P cycle, and glutamate metabolism (Sauer and Eikmanns, 2005; Chen et al., 2022b). To demonstrate this possibility, the expression of genes and activity of enzymes in the four metabolic pathways are measured in LTB4-R_CAZ_ and LTB4-S. Inactivation of the four metabolic pathways is documented in LTB4-R_CAZ_ compared with LTB4-S. Moreover, glucose is complemented to elevate the glucose level that is reduced in LTB4-R_CAZ_. The complementation promotes the glucose-mediated metabolic flux from glycolysis, the P cycle, and glutamate metabolism to purine metabolism. Therefore, the inefficient purine metabolism can be attributed to the depressed glucose. To further demonstrate that the inactivation of purine metabolism mediated by glucose is responsible for antibiotic resistance, exogenous glucose and guanosine are used to potentiate ceftazidime killing. The elevated killing efficacy is similar as the glucose did. Finally, the combination of ceftazidime with *pts* promoter is explored. Similar to ampicillin (Jiang et al., 2023), ceftazidime is bound to the *pts* promoter. Notably, our previous report showed that higher, unchanged, and lower expression of the four genes (*ptsI, ptsH, crr*, and *ptsG*) controlled by the promoter is detected in antibiotic-tolerant cells, at the switch point, and in antibiotic-resistant cells during chronic intermittent exposure to ampicillin (Jiang et al., 2023). LTB4-R_CAZ_ is a ceftazidime-resistant bacterium. Thus, a consistent result is a decrease in the expression of the four genes. Taken together, the present study identifies a glucose-mediated antibiotic resistance metabolic flux from glycolysis, the pyruvate cycle, and glutamate metabolism to purine metabolism, which is reversed by exogenous glucose and guanosine.

The core finding in the present study is that inefficient purine metabolism causes antibiotic resistance, which should be attributed to glucose depression. A recent metabolomics approach has shown that glucose and intermetabolites of purine metabolism are decreased simultaneously (Peng et al., 2015b; Zhang et al., 2019; Tang et al., 2022), but the relationship between them is absent. The present study provides robust proof that the intermetabolites of purine metabolism, such as guanosine, are a consequence of the depressed glucose. This finding not only clarifies the reason for reduced purine metabolism but also expands glucose-mediated antibiotic resistance mechanisms.

Equally importantly, following the first report that ampicillin binds with DNA, typically *pts* promoter, to manipulate the transition from tolerance to resistance in bacteria (Jiang et al., 2023), the present study further shows that ceftazidime has the same ability to bind to *pts* promoter. Since penicillin, ampicillin, and cephalosporin ceftazidime belong to β-lactams, this finding suggests that β-lactams have the ability to bind with *pts* promoters. In the evolution of antibiotic resistance, antibiotics are a trigger. Therefore, these reports on antibiotic-trigger resistance highlight the way to fully understand antibiotic resistance mechanisms.

Finally, the antibiotic resistance caused by the depressed glucose-mediated reduced purine metabolism can be reversed by not only triggering metabolite glucose but also lowering downstream metabolites such as guanosine. Metabolite-based metabolic reprogramming has been demonstrated to be effective in potentiating conventional antibiotics to kill antibiotic-resistant bacteria and promoting complement-mediated killing of serum-resistant bacteria (Cheng et al., 2019; Kou et al., 2022). More reprogramming metabolites are identified from upstream metabolites such as glucose and alanine (Peng et al., 2015b; Zhang et al., 2019; Chen et al., 2022a; Yin et al., 2022; Yang et al., 2023). The present study suggests that metabolic flux is an important clue to understanding the metabolic mechanisms underlying antibiotic resistance, which leads to downstream metabolites as reprogramming metabolites.

## Conclusion

The present study identifies a glucose-mediated antibiotic resistance metabolic flux from glycolysis, the pyruvate cycle, and glutamate metabolism to purine metabolism, which can be reversed by glucose and guanosine. In addition, the binding of ceftazidime with the *pts* promoter is revealed. These results highlight the need to fully understand antibiotic resistance mechanisms from a metabolic perspective.

## Data availability statement

The original contributions presented in the study are included in the article/Supplementary material, further inquiries can be directed to the corresponding authors.

## Author contributions

JX: Investigation, Methodology, Writing—original draft. S-wW: Methodology, Writing—original draft. YT: Investigation, Methodology, Writing—original draft. J-zY: Data curation, Investigation, Methodology, Writing—original draft. YL: Investigation, Resources, Writing—original draft. X-xP: Conceptualization, Writing—review and editing. L-fY: Conceptualization, Writing—review and editing. HL: Conceptualization, Writing—review and editing.
