# Supplementary material for: A glucose-mediated antibiotic resistance metabolic flux from glycolysis, the pyruvate cycle, and glutamate metabolism to purine metabolism
Source: Front Microbiol. 2023 Oct 17;14:1267729. doi: 10.3389/fmicb.2023.1267729 (PMC10616527; doi:10.3389/fmicb.2023.1267729)
Supplement: Supplementary file 1 [file Data_Sheet_1.docx]

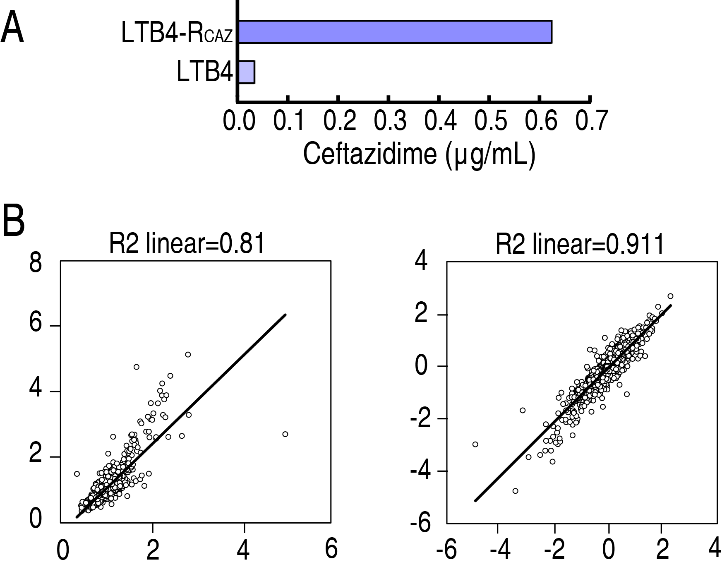


Supplementary Fig. 1. MIC and correlation coefficient between two biological replicates of LTB4-S and LTB4-R_CAZ_. A, MIC of LTB4-S and LTB4-R_CAZ_. B, Correlation between all proteins in LTB4-S and LTB4-R_CAZ_.

Supplementary Table 1. Primers used for qRT-PCR and *pts* promoter amplication.

|  | Primers used for qRT-PCR | |  |  |  |
| --- | --- | --- | --- | --- | --- |
| Genes | Primer | Sequence (5’-3’) | Genes | Primer | Sequence (5’-3’) |
| 16S rRNA | Forward | ACTGAGACACGGTCCAGACTCCTAC | *purN* | Forward | ATCGCCGCCTATCACCCTGA |
|  | Reverse | TTAACGTTCACACCTTCCTCCCTAC |  | Reverse | CGTCGCTGACAAAGTGAACG |
| *glk* | Forward | GTTCGATAGCCTGGAGTCGG | *purT* | Forward | CCGACAGCGTCATCGTAGAG |
|  | Reverse | AAAACGCCCAGCTGTGATTG |  | Reverse | AGCTCTACGCCAAACAGACC |
| *pgi* | Forward | GGTCGAAGAGGAGTTTGCCA | *purL* | Forward | CTTCCTGATCACCATCGGCG |
|  | Reverse | ATTTCACGCAGCAGGATGGA |  | Reverse | ATATCGCCGATGTAGCAGGC |
| *pfkA* | Forward | CGCTTGACTGAGATGGGGTT | *purM* | Forward | GCATGTACCACGGCGAAGAC |
|  | Reverse | GGCCTCTACAACGGTTTCCA |  | Reverse | CGCTGACCTCCAGGATCTTG |
| *pfkB* | Forward | CTTGGTGGTGAGTGGGAGTC | *purK* | Forward | ACGAACCCCAGCTTGATGC |
|  | Reverse | CGCCAGCTCCTTTTGATTGG |  | Reverse | CTCCTGCAGAAAGCTCTCCG |
| *fbaA* | Forward | TCCTCCCACATGATCGACCT | *purE* | Forward | GAAGAAGCTGGAGATGGTGC |
|  | Reverse | GTCCAGATGGCTGTTGTCCA |  | Reverse | GCTGACCCAGCCAAAGGTAC |
| *gapA* | Forward | AACGATCTGCTGGATGCCAA | *purC* | Forward | CCTGCTGTCCGATACCGATG |
|  | Reverse | CAACAACGTCAACGCCGATT |  | Reverse | TCTTCAATGCCCAGACGCTT |
| *ETAE_2957* | Forward | GTTATGGATGCGTTCGGCAC | *purB2* | Forward | CGACCGTCGGCAAAGAGTTC |
|  | Reverse | AGTCCAGAACGGTCAGCTTG |  | Reverse | GTGGTGTAGGGGTTCCACTG |
| *gpmB* | Forward | TTCAGGGACAGTCGAACAGC | *purH* | Forward | GAGGAGCCACAGGTTGAAGC |
|  | Reverse | CTGGCGATCACGTGGGTAAT |  | Reverse | TGGTAAGCGCGCTCATAGGC |
| *ETAE_2321* | Forward | AGCTGAACCAGATAGGCAGC | *guaB* | Forward | GCTGATCGACTCCTCTCACG |
|  | Reverse | CTATCCACGGTTTCACCGCT |  | Reverse | ATACCCACTTTGACGGCGTT |
| *pykF* | Forward | TCATGCAGAGCCGTATCGAC | *guaA* | Forward | GGCGATGGCCGTAAGTATGA |
|  | Reverse | GATCATCGCATCCGGGAAGT |  | Reverse | TCGTTGATGATGCGGTTGGA |
| *gltB* | Forward | GCCTGTTCCACCAACGTTTC | *ETAE_1034* | Forward | CGAACTATGTCCCCGGTACG |
|  | Reverse | GTTTCGTTGACAAAGGGGGC |  | Reverse | TCCTGCCGATTGAGGTTCAC |
| *gltD* | Forward | GTTTGACGCGGTCTTTCTCG | *ptsI* | Forward | AAGGCGCTTCTTCTGAAAGA |
|  | Reverse | GACGTAGGGGGTCTGAGGAT |  | Reverse | TGCTCAAGCTCTTCATCTTC |
| *glnA* | Forward | AGGGTAAAGAGCAGCACGTC | *ptsH* | Forward | CAAAGCATTCGCTTCTGACA |
|  | Reverse | CATTGATGCCTTTCCAGCCG |  | Reverse | GGCGTTCTGCTCATCTTCAC |
| *ETAE_2409* | Forward | CGAATACATGGTCGCCTCTG | *crr* | Forward | ATCGGTAAAATCTTTGAAACT |
|  | Reverse | TCGATAAACGAGTCCGGGCG |  | Reverse | ATCAGCTCCTTGATCTCATC |
| *purD* | Forward | ATCGTTATGGTCGACGGCGA | *ptsG* | Forward | TGATGCTGCCGGTATCAGTC |
|  | Reverse | TGCCATACCGCGTACGGTTG |  | Reverse | GCTTGGAAGCAATCTCTTCC |
|  |  |  |  |  |  |
|  | Primers for *pts* promoter | |  |  |  |
| Genes | Primer | Sequence (5’-3’) |  |  |  |
| *pts* promoter | Forward | GCGGCGAATGCGATGGCATG |  |  |  |
|  | Reverse | TAAAATAGTTGGGGAAACCT |  |  |  |

Supplementary Tables 2. Identification of significantly up-regulated proteins involved in *E. tarda* in response of ceftazidime resistance using iTRAQ labeling analysis.

| Accession | Description | *Edwardsiella tarda*  Genename | Coverage(99%) | Matched  peptides | Repeat 1 (log2 ratio) | Repeat 2 (log2 ratio) | ave (log2 ratio) | p-value |
| --- | --- | --- | --- | --- | --- | --- | --- | --- |
| UPI0000006216 | DNA-binding protein Fis | *fis* | 28.57 | 2 | 2.27 | 2.68 | 2.48 | 0.0010 |
| UPI0001E35535 | 50S ribosomal protein L36 | *rpmJ* | 21.05 | 1 | 1.81 | 2.28 | 2.04 | 0.0033 |
| UPI0000002502 | Long polar fimbria protein A | A9797_05070 | 40.11 | 4 | 1.96 | 2.03 | 1.99 | 0.0094 |
| UPI0002C0B5A2 | TonB-dependent hemin, ferrichrome receptor | ETAC_08250 | 9.62 | 5 | 1.69 | 1.82 | 1.75 | 0.0036 |
| UPI0002C143FE | Vibrioferrin receptor PvuA | ETAC_09020 | 2.02 | 1 | 1.49 | 1.94 | 1.72 | 0.0089 |
| UPI0001BD0221 | Putrescine importer | A9797_04670 | 1.76 | 1 | 1.54 | 1.64 | 1.59 | 0.0010 |
| UPI0001BD05D9 | Sulfur acceptor protein SufE | A9797_03610 | 13.51 | 2 | 1.40 | 1.67 | 1.53 | 0.0036 |
| UPI0002C089AA | Uncharacterized protein | ETAC_05735 | 2.57 | 1 | 1.59 | 1.46 | 1.53 | 0.0025 |
| UPI0002C0EC43 | Vibrioferrin amide bond forming protein PvsD | ETAC_09005 | 9.48 | 5 | 1.59 | 1.34 | 1.47 | 0.0083 |
| UPI0001BD068C | Protein RecA | *recA* | 68.56 | 20 | 1.53 | 1.38 | 1.46 | 0.0097 |
| UPI0000002503 | Fimbrial protein | A9797_05075 | 17.98 | 4 | 1.27 | 1.49 | 1.38 | 0.0027 |
| UPI0001BD086D | Hemin transport protein HmuS | ETAC_08255 | 62.61 | 13 | 1.48 | 1.24 | 1.36 | 0.0055 |
| UPI0002C0D08C | Endonuclease III | *nth* | 5.63 | 3 | 1.08 | 1.47 | 1.28 | 0.0089 |
| UPI0001BD0550 | Mutator MutT protein | ETAC_03135 | 9.56 | 1 | 1.23 | 1.27 | 1.25 | 0.0006 |
| UPI0001A5A717 | 50S ribosomal protein L23 | *rplW* | 62.00 | 9 | 1.10 | 1.34 | 1.22 | 0.0036 |
| UPI0001BD07DF | GTP 3',8-cyclase | *moaA* | 8.84 | 3 | 1.40 | 1.04 | 1.22 | 0.0091 |
| UPI0001A5A704 | 30S ribosomal protein S13 | *rpsM* | 62.71 | 11 | 1.09 | 1.28 | 1.18 | 0.0044 |
| UPI0001BD0567 | Hypoxanthine phosphoribosyltransferase | A9797_03375 | 27.53 | 4 | 1.07 | 1.26 | 1.16 | 0.0067 |
| UPI0001BD0B33 | 30S ribosomal protein S14 | *rpsN* | 37.62 | 7 | 1.17 | 1.14 | 1.15 | 0.0016 |
| UPI0002C1011A | Uncharacterized protein | ETAC_04920 | 23.88 | 5 | 1.09 | 1.20 | 1.15 | 0.0035 |
| UPI0002C042F3 | tRNA 2-thiocytidine biosynthesis protein TtcA | *ttcA* | 22.26 | 6 | 1.07 | 1.09 | 1.08 | 0.0000 |
| UPI0002C14047 | Protease | ETAC_05075 | 20.22 | 9 | 1.02 | 1.09 | 1.06 | 0.0043 |
| UPI0002C0B709 | Protease SohB | A9797_07460 | 12.93 | 3 | 1.09 | 1.00 | 1.04 | 0.0009 |
| UPI0001A5A707 | L-threonine dehydrogenase | A9797_03865 | 44.07 | 5 | 1.02 | 0.98 | 1.00 | 0.0010 |
| UPI0002C0A4EE | Peptidoglycan glycosyltransferase | ETAC_06265 | 2.23 | 2 | 0.85 | 1.15 | 1.00 | 0.0093 |
| UPI0001BD0806 | Small-conductance mechanosensitive channel protein | A9797_14700 | 39.86 | 9 | 1.01 | 0.94 | 0.97 | 0.0020 |
| UPI0002C07C58 | Ribonucleoside-diphosphate reductase | A9797_11115 | 56.84 | 38 | 1.05 | 0.89 | 0.97 | 0.0075 |
| UPI0002C0EF32 | NADH dehydrogenase | ETAC_09840 | 40.55 | 15 | 0.98 | 0.95 | 0.97 | 0.0016 |
| UPI0002C1342C | 50S ribosomal protein L3 | *rplC* | 69.38 | 14 | 0.98 | 0.93 | 0.96 | 0.0026 |
| UPI0001BD09C6 | Uncharacterized protein | A9797_09170 | 36.96 | 5 | 0.96 | 0.91 | 0.94 | 0.0024 |
| UPI0001BD0469 | OmpA family lipoprotein | A9797_00085 | 26.94 | 4 | 0.89 | 0.93 | 0.91 | 0.0076 |
| UPI0001BD010C | Carboxy-S-adenosyl-L-methionine synthase | *cmoA* | 5.08 | 2 | 1.02 | 0.79 | 0.91 | 0.0065 |
| UPI0001BD02C7 | UPF0597 protein A9797_01950 | A9797_01950 | 2.51 | 1 | 0.92 | 0.89 | 0.91 | 0.0087 |
| UPI0002C0DE01 | Uncharacterized protein | ETAC_01215 | 11.56 | 3 | 0.97 | 0.82 | 0.90 | 0.0033 |
| UPI0002C0DA4C | Ribonucleotide-diphosphate reductase subunit beta | A9797_11120 | 38.03 | 14 | 0.93 | 0.85 | 0.89 | 0.0008 |
| UPI0001A5A959 | 30S ribosomal protein S16 | *rpsP* | 71.95 | 6 | 0.84 | 0.93 | 0.88 | 0.0067 |
| UPI0001BD0247 | Adenylosuccinate synthetase | *purA* | 53.47 | 20 | 0.75 | 1.01 | 0.88 | 0.0091 |
| UPI0001BD0607 | Sec-independent protein translocase protein TatA | *tatA* | 52.81 | 4 | 0.93 | 0.75 | 0.84 | 0.0054 |
| UPI0001BD0AE0 | Peptide deformylase | *def* | 53.22 | 6 | 0.79 | 0.88 | 0.84 | 0.0015 |
| UPI0001BD0265 | 50S ribosomal protein L21 | *rplU* | 69.90 | 8 | 0.84 | 0.81 | 0.83 | 0.0039 |
| UPI0002C12139 | Uncharacterized protein | ETAC_04715 | 38.02 | 5 | 0.94 | 0.71 | 0.83 | 0.0088 |
| UPI0001BD061C | 50S ribosomal protein L11 | *rplK* | 83.80 | 11 | 0.73 | 0.90 | 0.81 | 0.0099 |
| UPI0001BD03C6 | Recombination-associated protein RdgC | *rdgC* | 49.50 | 13 | 0.79 | 0.83 | 0.81 | 0.0026 |
| UPI0001E3551B | Uncharacterized protein | ETAC_15040 | 23.53 | 3 | 0.86 | 0.76 | 0.81 | 0.0027 |
| UPI0002C1061B | Ribosome-binding factor A | *rbfA* | 59.85 | 7 | 0.70 | 0.91 | 0.81 | 0.0077 |
| UPI0001BD07DC | Molybdenum cofactor biosynthesis protein E Molybdopterin converting factor subunit 2 | ETAF_2116 | 20.67 | 3 | 0.74 | 0.86 | 0.80 | 0.0061 |
| UPI0002C091C5 | 1-acyl-sn-glycerol-3-phosphate acyltransferase | A9797_00930 | 14.57 | 3 | 0.69 | 0.90 | 0.80 | 0.0079 |
| UPI0001BD02ED | ATP-dependent Clp protease ATP-binding subunit ClpX | *clpX* | 73.29 | 22 | 0.78 | 0.82 | 0.80 | 0.0005 |
| UPI0002C0B8B4 | ATP-dependent RNA helicase SrmB | *srmB* | 42.63 | 16 | 0.87 | 0.70 | 0.79 | 0.0052 |
| UPI0001BD0D2C | Uncharacterized protein | A9797_13955 | 37.88 | 2 | 0.82 | 0.74 | 0.78 | 0.0023 |
| UPI0001BD0680 | 50S ribosomal protein L19 | *rplS* | 62.61 | 7 | 0.80 | 0.74 | 0.77 | 0.0029 |
| UPI0002C048F2 | Amino acid ABC transporter substrate-binding protein | A9797_07410 | 59.38 | 13 | 0.75 | 0.77 | 0.76 | 0.0005 |
| UPI0001BD027F | Protein translocase subunit SecD | *secD* | 37.54 | 21 | 0.73 | 0.78 | 0.75 | 0.0012 |
| UPI0001BD063B | Elongation factor Ts | *tsf* | 73.33 | 24 | 0.78 | 0.71 | 0.75 | 0.0019 |
| UPI0001A5A8C6 | Iron-sulfur cluster assembly scaffold protein IscU | A9797_13980 | 71.88 | 6 | 0.82 | 0.66 | 0.74 | 0.0063 |
| UPI0001BD0763 | ATP-dependent RNA helicase RhlE | *rhlE* | 30.00 | 10 | 0.74 | 0.75 | 0.74 | 0.0049 |
| UPI0002C0C21F | tRNA-2-methylthio-N(6)-dimethylallyladenosine synthase | *miaB* | 36.50 | 15 | 0.71 | 0.76 | 0.73 | 0.0098 |
| UPI0001BD0126 | Uncharacterized protein | A9797_01270 | 12.50 | 2 | 0.65 | 0.80 | 0.73 | 0.0049 |
| UPI0001BD0D31 | Cysteine desulfurase IscS | *iscS* | 77.23 | 25 | 0.69 | 0.75 | 0.72 | 0.0006 |
| UPI0001BD0B32 | 30S ribosomal protein S8 | *rpsH* | 61.54 | 7 | 0.63 | 0.80 | 0.71 | 0.0063 |
| UPI0001A59DB2 | 50S ribosomal protein L27 | A9797_01825 | 63.53 | 7 | 0.71 | 0.71 | 0.71 | 0.0094 |
| UPI0001A5A681 | Cold shock protein CspC | A9797_07480 | 95.65 | 6 | 0.65 | 0.76 | 0.71 | 0.0049 |
| UPI0002C08297 | 3-oxoacyl-[acyl-carrier-protein] synthase 2 | A9797_10065 | 34.87 | 9 | 0.68 | 0.71 | 0.70 | 0.0081 |
| UPI0001BD04B5 | Iron-sulfur cluster carrier protein | A9797_05825 | 25.95 | 8 | 0.72 | 0.67 | 0.69 | 0.0067 |
| UPI0001BD02F1 | Peptidylprolyl isomerase | A9797_04920 | 46.01 | 21 | 0.63 | 0.76 | 0.69 | 0.0089 |
| UPI0002C0AA26 | Tol-Pal system protein TolQ | *tolQ* | 24.23 | 4 | 0.69 | 0.69 | 0.69 | 0.0001 |
| UPI0001BD03FD | outer membrane protein assembly factor BamC | *bamC* | 43.93 | 11 | 0.70 | 0.67 | 0.69 | 0.0005 |
| UPI0001BD0C82 | Multidrug resistance protein A | A9797_13705 | 30.95 | 9 | 0.62 | 0.73 | 0.68 | 0.0029 |
| UPI0001BD027D | Queuine tRNA-ribosyltransferase | *tgt* | 42.22 | 15 | 0.65 | 0.71 | 0.68 | 0.0092 |
| UPI0002C04DEE | Acetyltransferase component of pyruvate dehydrogenase complex | *aceF* | 35.53 | 19 | 0.67 | 0.67 | 0.67 | 0.0004 |
| UPI0002C14462 | Magnesium/cobalt efflux protein | A9797_14190 | 11.45 | 4 | 0.65 | 0.68 | 0.66 | 0.0026 |
| UPI0001A599D1 | 50S ribosomal protein L1 | A9797_00750 | 79.49 | 20 | 0.66 | 0.66 | 0.66 | 0.0017 |
| UPI0002C081C9 | Ribosomal RNA small subunit methyltransferase C | *rsmC* | 36.71 | 8 | 0.68 | 0.63 | 0.66 | 0.0005 |
| UPI0001A5A2A4 | ABC-F family ATPase | A9797_12405 | 28.44 | 14 | 0.63 | 0.67 | 0.65 | 0.0053 |
| UPI0001BD0338 | TIGR00153 family protein | A9797_02360 | 24.78 | 6 | 0.65 | 0.64 | 0.64 | 0.0000 |
| UPI0001BD0798 | Low affinity potassium transport system protein kup | *trkD* | 3.70 | 2 | 0.59 | 0.69 | 0.64 | 0.0026 |
| UPI0001BD0B7C | D-alanyl-D-alanine carboxypeptidase | ETAC_12720 | 42.68 | 13 | 0.67 | 0.59 | 0.63 | 0.0026 |
| UPI0002C06396 | Glycerol-3-phosphate dehydrogenase | *glpD* | 70.72 | 32 | 0.58 | 0.68 | 0.63 | 0.0041 |
| UPI0001E3552E | Nicotinate phosphoribosyltransferase | *pncB* | 16.21 | 5 | 0.65 | 0.60 | 0.63 | 0.0021 |
| UPI0002C07A4F | Lipoyl synthase | *lipA* | 30.53 | 8 | 0.68 | 0.57 | 0.62 | 0.0041 |
| UPI0001A5A70A | 50S ribosomal protein L6 | *rplF* | 75.14 | 11 | 0.63 | 0.61 | 0.62 | 0.0032 |
| UPI0002C0E3F3 | Vibrioferrin ligase/carboxylase protein PvsA | ETAC_08990 | 10.28 | 3 | 0.65 | 0.59 | 0.62 | 0.0074 |
| UPI0001BD0496 | RNA polymerase-associated protein RapA | *rapA* | 23.86 | 14 | 0.60 | 0.63 | 0.62 | 0.0083 |
| UPI0001BD0642 | Outer membrane protein assembly factor BamA | *bamA* | 38.49 | 30 | 0.66 | 0.56 | 0.61 | 0.0034 |
| UPI0002C113C3 | Signal peptidase I | ETAC_13165 | 27.16 | 7 | 0.54 | 0.68 | 0.61 | 0.0076 |
| UPI0002C0D2E9 | 3-oxoacyl-ACP reductase | *fabG* | 54.92 | 11 | 0.65 | 0.55 | 0.60 | 0.0042 |
| UPI0002C0EFF6 | Protein FdhE homolog | *fdhE* | 11.04 | 2 | 0.59 | 0.61 | 0.60 | 0.0082 |
| UPI0001BD0904 | 50S ribosomal protein L3 glutamine methyltransferase | *prmB* | 24.19 | 6 | 0.56 | 0.63 | 0.59 | 0.0021 |
| UPI0002C15174 | Protein YceD | A9797_10100 | 35.84 | 7 | 0.61 | 0.58 | 0.59 | 0.0016 |
| UPI0001BD011D | Adenine DNA glycosylase | ETAC_01115 | 4.14 | 1 | 0.53 | 0.65 | 0.59 | 0.0089 |
| UPI0001A5A41D | Ribosome-binding ATPase YchF | *ychF* | 38.02 | 11 | 0.60 | 0.57 | 0.59 | 0.0020 |

Supplementary Tables 3. Identification of significantly down-regulated proteins involved in *E. tarda* in response of ceftazidime resistance using iTRAQ labeling analysis.

| Accession | Description | *Edwardsiella tarda*  Genename | Coverage(99%) | Matched  peptides | Repeat 1 (log2 ratio) | Repeat 2 (log2 ratio) | ave (log2 ratio) | p-value |
| --- | --- | --- | --- | --- | --- | --- | --- | --- |
| UPI0001BD0A5B | Phospho-2-dehydro-3-deoxyheptonate aldolase | A9797_12630 | 28.53 | 7 | -0.52 | -0.66 | -0.59 | 0.0077 |
| UPI0001BD0A06 | Glutathione S-transferase | A9797_15415 | 56.16 | 8 | -0.53 | -0.66 | -0.59 | 0.0061 |
| UPI0001BD0A1F | Uncharacterized protein | A9797_09265 | 14.77 | 5 | -0.55 | -0.64 | -0.60 | 0.0035 |
| UPI0002C04156 | ADP-ribose pyrophosphatase NudF | *nudF* | 43.06 | 6 | -0.56 | -0.64 | -0.60 | 0.0023 |
| UPI0002C0BE05 | Isoaspartyl dipeptidase | ETAC_06440 | 49.74 | 17 | -0.59 | -0.62 | -0.61 | 0.0004 |
| UPI0002C0E859 | Protein lysine acetyltransferase | ETAC_02705 | 12.75 | 9 | -0.59 | -0.64 | -0.61 | 0.0037 |
| UPI00000DC6E1 | Translational regulator CsrA | *csrA* | 81.97 | 8 | -0.55 | -0.68 | -0.62 | 0.0080 |
| UPI0001E3543B | Fumarate reductase subunit C | *frdC* | 31.06 | 6 | -0.58 | -0.68 | -0.63 | 0.0049 |
| UPI0002C0EC54 | Phosphoribosylformylglycinamidine synthase | *purL* | 13.90 | 14 | -0.57 | -0.69 | -0.63 | 0.0070 |
| UPI0001BD0AB7 | Citrate synthase | A9797_12760(CIT) | 61.83 | 22 | -0.60 | -0.67 | -0.63 | 0.0015 |
| UPI0002C10F2B | Fumarate reductase flavoprotein subunit | A9797_01560 | 64.61 | 31 | -0.61 | -0.67 | -0.64 | 0.0034 |
| UPI0002C1010E | Bifunctional indole-3-glycerol phosphate synthase/phosphoribosylanthranilate isomerase | ETAC_07185 | 37.34 | 14 | -0.66 | -0.63 | -0.65 | 0.0027 |
| UPI0001BD0776 | Uncharacterized protein | A9797_14510 | 26.87 | 7 | -0.73 | -0.59 | -0.66 | 0.0097 |
| UPI0002C0F025 | Xanthine dehydrogenase subunit XdhA | ETAC_02855 | 23.98 | 16 | -0.57 | -0.75 | -0.66 | 0.0084 |
| UPI0001BD00C8 | Nitrate reductase Z subunit alpha | ETAC_01055 | 32.87 | 31 | -0.66 | -0.68 | -0.67 | 0.0043 |
| UPI0002C0B26A | Protein EsaQ | ETAC_04195 | 24.01 | 5 | -0.74 | -0.60 | -0.67 | 0.0082 |
| UPI0001BD0D3F | Chaperone protein ClpB | *clpB* | 71.18 | 64 | -0.60 | -0.77 | -0.68 | 0.0098 |
| UPI0002C0C49D | Guanine deaminase | ETAC_04090 | 34.59 | 13 | -0.71 | -0.66 | -0.69 | 0.0034 |
| UPI0002C0D561 | Glucose-1-phosphatase/inositol phosphatase | ETAC_08070 | 37.86 | 12 | -0.70 | -0.69 | -0.69 | 0.0000 |
| UPI0001BD0355 | Uncharacterized protein | A9797_05125 | 10.00 | 1 | -0.72 | -0.67 | -0.70 | 0.0020 |
| UPI0001BD08A3 | Hydrogenase maturation factor HoxO | ETAC_14425 | 61.83 | 6 | -0.72 | -0.71 | -0.71 | 0.0034 |
| UPI0002C09F68 | Transcriptional regulator | A9797_00540 | 3.31 | 1 | -0.62 | -0.81 | -0.71 | 0.0074 |
| UPI0000004ABC | Phosphate-binding protein PstS | *pstS* | 51.16 | 14 | -0.72 | -0.71 | -0.72 | 0.0005 |
| UPI0002C05420 | Phosphoribosylamine--glycine ligase | *purD* | 27.53 | 8 | -0.72 | -0.72 | -0.72 | 0.0094 |
| UPI0001BD0C90 | Formate dehydrogenase iron-sulfur subunit | A9797_16530 | 14.83 | 5 | -0.69 | -0.77 | -0.73 | 0.0012 |
| UPI0001BD00FE | Uncharacterized protein | A9797_06825 | 2.72 | 1 | -0.72 | -0.79 | -0.75 | 0.0059 |
| UPI0002C13CD9 | Chondroitin sulfate ABC lyase | ETAC_14050 | 26.03 | 21 | -0.69 | -0.81 | -0.75 | 0.0042 |
| UPI0001BD074B | ATP-dependent dethiobiotin synthetase BioD | *bioD* | 7.24 | 1 | -0.68 | -0.83 | -0.76 | 0.0084 |
| UPI0001BD0BEC | CheY-like receiver | ETAC_12890 | 8.08 | 2 | -0.71 | -0.81 | -0.76 | 0.0020 |
| UPI0002C14F99 | Chondroitin sulfate ABC lyase | ETAC_14055 | 37.04 | 30 | -0.79 | -0.79 | -0.79 | 0.0055 |
| UPI0002C0AC0B | TIGR00156 family protein | A9797_02110 | 54.14 | 10 | -0.83 | -0.76 | -0.79 | 0.0084 |
| UPI0002C0CEC9 | Molybdenum ABC transporter substrate-binding protein | ETAC_04410 | 39.50 | 6 | -0.88 | -0.73 | -0.80 | 0.0076 |
| UPI0002C13650 | Putative nickel-responsive regulator | ETAC_01100 | 18.37 | 2 | -0.82 | -0.81 | -0.81 | 0.0005 |
| UPI0002C0F1F1 | Phage-related tail protein | ETAC_16395 | 8.70 | 1 | -0.78 | -0.90 | -0.84 | 0.0080 |
| UPI0001BD0A7E | Chlorohydrolase | A9797_15645 | 52.26 | 19 | -0.87 | -0.87 | -0.87 | 0.0080 |
| UPI0002C04CF0 | Autotransporter outer membrane beta-barrel domain-containing protein | A9797_14515 | 9.77 | 6 | -0.86 | -0.98 | -0.92 | 0.0029 |
| UPI0002C05524 | 5-hydroxyisourate hydrolase | ETAC_05035 | 16.30 | 2 | -0.92 | -0.94 | -0.93 | 0.0003 |
| UPI0001BD08CA | Integration host factor subunit alpha | *ihfA* | 70.41 | 10 | -0.89 | -0.97 | -0.93 | 0.0024 |
| UPI0001BD095A | Peptidase T | *pepT* | 32.28 | 13 | -0.98 | -0.92 | -0.95 | 0.0039 |
| UPI0001BD0A7A | Guanine deaminase | A9797_15625 | 38.81 | 15 | -0.98 | -0.97 | -0.98 | 0.0091 |
| UPI0002C0E239 | Phage tail tape measure protein, TP901 family | ETAC_09520 | 1.61 | 1 | -0.97 | -1.07 | -1.02 | 0.0073 |
| UPI0002C13B66 | 4-deoxy-L-threo-5-hexosulose-uronate ketol-isomerase | *kduI* | 27.70 | 5 | -1.02 | -1.05 | -1.03 | 0.0013 |
| UPI0002C0F092 | Acetylornithine aminotransferase | *argD* | 18.56 | 5 | -0.89 | -1.19 | -1.04 | 0.0087 |
| UPI0001BD0420 | Thioredoxin 2 | A9797_02855 | 8.39 | 1 | -0.90 | -1.19 | -1.04 | 0.0091 |
| UPI0002C0A1DA | Ribose/xylose/arabinose/galactoside ABC-type transport system, periplasmic sugar binding protein | ETAC_16020 | 71.02 | 18 | -1.11 | -0.97 | -1.04 | 0.0073 |
| UPI0001BD0C7E | DNA starvation/stationary phase protection protein | A9797_13690 | 70.11 | 11 | -0.98 | -1.12 | -1.05 | 0.0069 |
| UPI0001BD0775 | Choline-sulfatase | ETAC_14035 | 52.94 | 21 | -1.15 | -1.00 | -1.08 | 0.0044 |
| UPI0002C1186B | Glutamate decarboxylase | ETAC_13810 | 73.49 | 25 | -1.05 | -1.15 | -1.10 | 0.0038 |
| UPI0002C0D73D | HTH-type transcriptional repressor NanR | *nanR* | 3.42 | 1 | -1.03 | -1.19 | -1.11 | 0.0091 |
| UPI0001BD0BFA | N5-carboxyaminoimidazole ribonucleotide mutase | *purE* | 29.59 | 4 | -1.10 | -1.15 | -1.13 | 0.0008 |
| UPI0001BD039E | Ribosome hibernation protein YhbH | A9797_02520 | 37.89 | 3 | -1.07 | -1.19 | -1.13 | 0.0013 |
| UPI0000552F19 | Transcriptional regulator | A9797_04305 | 6.52 | 1 | -1.14 | -1.21 | -1.17 | 0.0036 |
| UPI0002C0FF85 | p-hydroxybenzoic acid efflux pump subunit AaeA | *aaeA* | 4.50 | 1 | -1.25 | -1.15 | -1.20 | 0.0065 |
| UPI0002C102F7 | Permease | A9797_15620 | 4.40 | 1 | -1.09 | -1.46 | -1.28 | 0.0093 |
| UPI0002C100A9 | Glutamine ABC transporter periplasmic protein | *glnH* | 48.79 | 10 | -1.26 | -1.34 | -1.30 | 0.0004 |
| UPI0001BD03BE | Uncharacterized protein | A9797_05235 | 5.31 | 1 | -1.37 | -1.44 | -1.40 | 0.0066 |
| UPI0002C06E07 | Lysine-specific permease | ETAC_14800 | 2.14 | 1 | -1.50 | -1.67 | -1.59 | 0.0030 |
| UPI0002C0CFAC | Hydroxymethylglutaryl-CoA lyase | ETAC_09305 | 3.66 | 1 | -1.59 | -1.68 | -1.64 | 0.0046 |
| UPI0002C0F802 | NADH:flavin oxidoreductase | A9797_04795 | 1.17 | 1 | -2.34 | -2.21 | -2.28 | 0.0002 |
